# Distribution of distinct subsets of circulating T follicular helper cells in Kawasaki disease

**DOI:** 10.1186/s12887-019-1412-z

**Published:** 2019-01-31

**Authors:** Meng Xu, Yanfang Jiang, Jinghua Wang, Deying Liu, Shaofeng Wang, Huanfa Yi, Sirui Yang

**Affiliations:** 1 Department of Pediatric Rheumatology and Allergy, The First Hospital of Jilin University, Changchun, 130021 China; 2 Genetic Diagnosis Center, The First Hospital of Jilin University, Changchun, 130021 China; 3 Key Laboratory of Zoonoses Research, Ministry of Education, The First Hospital of Jilin University, Changchun, 130021 China; 4 Jiangsu Co-innovation Center for Prevention and Control of Important Animal Infectious Diseases and Zoonoses, Yangzhou, 225009 China; 5 The Bethune Institute of Epigenetic Medicine, The First Hospital of Jilin University, Changchun, 130021 China; 6 Central Laboratory of the Eastern Division, The First Hospital of Jilin University, Changchun, 130021 China

**Keywords:** Kawasaki disease, cTfh cells, Coronary artery lesions

## Abstract

**Background:**

Kawasaki disease (KD) is an acute febrile vasculitis that primarily affects children. Previous studies have shown that both innate and adapt immune systems are involved in the immunopathogenesis of KD. The following study analyzes the distribution of the subsets of Circulating T follicular helper cells (cTfh cells) in KD patients with and without coronary artery lesions (CALs).

**Methods:**

Twenty KD patients and fifteen healthy sex- and age- matched children were enrolled. Patients were divided into two groups depending on CALs. Blood samples were collected respectively before and after intravenous immunoglobulin (IVIG) administration. Circulating Tfh cells were categorized into three subsets by flow cytometry including cTfh1 (CXCR3 + CCR6-), cTfh2 (CXCR3-CCX6-) and cTfh17 (CXCR3-CCR6+) cells in circulating CD3 + CD4 + CXCR5 + CD45RA- T cells. Cytometric bead arrays were used to analyze the level of IFN-γ, IL-4 and IL-17A.

**Results:**

We found that frequency of cTfh2 cells was significantly elevated in KD patients before IVIG administration with low expression of cTfh1 cells, where the ratio of cTfh2 + cTfh17/cTfh1 significantly increased. Levels of IFN-γ, IL-4 and IL-17A in KD were significantly higher compared to controls. Further analysis showed that cTfh1 cells were negatively correlated with serum CRP, whereas cTfh2 cells were positively correlated with serum CRP and ESR. Comparison of different groups showed that frequency of cTfh1 cells in CALs+ group were significantly lower compared to CALs- group. In contrast, cTfh2 cells in CALs+ group significantly increased. After IVIG administration, frequency of cTfh2 cells and the ratio significantly decreased while the frequency of cTfh1 cells significantly increased. Meanwhile, all levels of cytokines decreased.

**Conclusions:**

Our data demonstrated that cTfh1 and cTfh2 cells participate in the pathogenesis of KD, and that the two subsets might be associated with CALs.

## Background

Kawasaki disease (KD) is a self-limited systemic vasculitis of small- and media-sized arteries that predominately affects children under the age of 5 years. In the absence of specific indicators, the diagnosis still rests on the clinical symptoms and full exclusion of other possibilities. The conventional diagnostic criteria of KD upon clinical symptoms include fever persisting for at least 5 days, erythema and desquamation of extremity, polymorphic rash, bilateral conjunctivitis, oral changes and cervical lymphadenopathy (diameter ≥ 1.5 cm) [1]. The most serious complications of KD are coronary artery lesions (CALs), which are thought to be the leading cause of acquired heart disease in childhood. With the discovery and clinical application of intravenous immunoglobulin (IVIG) therapy, the incidence of coronary artery disease is significantly reduced. Since it was first reported in 1967, Kawasaki disease has been widely studied, nonetheless it’s cause remains unclear. It is commonly thought to be triggered by a pathogen that causes a series of aberrant immune responses. Studies related to immune response have suggested that both innate and adaptive immune systems are involved, leading to impaired immune homeostasis [2, 3]. Consequently, understanding the immunopathogenesis of KD is beneficial for the formulation of treatment strategies for KD patients.

Several studies have reported the role of peripheral CD4+ T cell in KD, nonetheless, the results remain controversial [4–6]. As a distinct subset of CD4+ T cells, follicular T helper (Tfh) cells have been initially described in the germinal centers (GC) of secondary lymphoid tissues, and can be identified by a biomarker named CXC-chemokine receptor 5 (CXCR5) and by their ability to provide help in selecting high-affinity B cells [7, 8]. On account of poor access to tissue samples, Tfh cells in peripheral blood are established, and are named circulating Tfh cells [9]. Given circulating Tfh (cTfh) cells share the immunophenotype and functional nature of GC Tfh cells, cTfh cells can be categorized into distinct subsets by differential expression of biomarker such as CXCR3 and CCR6. CXCR3 is required for IFN-gamma-producing T cells (Th1 cells), while CCR6 is essential for IL-17-producing T cells (Th17 cells) [10]. Three subpopulations of cTfh cells are defined on the basis of the combination of CXCR3 and CCR6: CXCR3 + CCR6- cells share properties with Th1 cells, CXCR3-CCR6- cells share properties with Th2 cells, and CXCR3-CCR6+ cells share properties with Th17 cells [11]. For this reason, they are respectively called cTfh1, cTfh2 and cTfh17 cells. According to the ability in inducing B cells to proliferate, producing immunoglobulins and switching immunoglobulin isotypes when co-cultured with B cells, these three subsets can be divided into efficient cells (cTfh2 and cTfh17 cells) and non-efficient cells (cTfh1 cells) [12]. Furthermore, cTfh2 cells contribute to the production of IgG and IgE, whereas cTfh17 cells promote IgG and IgA secretion [13, 14]. In contrast, cTfh1 cells have impact on T cells accumulation in mice, but not in human [15, 16]. Current understanding of aberrant cTfh response has shown that imbalance of cTfh-cell subsets, which represents over-expression in cTfh2 or cTfh17 cells and under-expression in cTfh1 cells, is related to multiple diseases. However, the association between these three subsets and Kawasaki disease remains to be investigated.

CTfh cells are considered as a memory compartment of germinal center Tfh cells [13]. It is conventionally believed that CD4+ memory T cell contribute to endow the host with more rapid and efficient abilities in secondary immune responses. Recurring inflammation and the progression of disease are suspected to be the consequence of CD4+ memory T cell dysregulation [17]. Thus, the self-limited property and low recurrence incidence of KD have suggested that circulating memory cells are involved in this process. In the present study, we focus on the subsets of circulating Tfh cells in KD patients including those with and without coronary artery lesions, where we analyze their association with clinical parameters. Our results are helpful to further understand the immunological pathogenesis of KD.

## Methods

### Patients

A total of 20 first-treated KD patients and 15 sex and age-matched healthy controls were enrolled at the Department of Pediatrics, The First Hospital of Jilin University, China, from January to December 2017. The diagnostic criterion was made upon detailed physical and laboratory tests according to 2017 American Heart association (AHA) clinical statement and guidelines [1]. Z-score corrected by BSA and provided from Dallaire et al. was used to evaluate the CALs, which was defined as Z-score ≥ 2 [18]. We used Haycock et al.’s equation to compute BSA [19]. Based on the observation under echocardiography and Z-score, patients were categorized into two groups, CALs+ group and CALs- group. Six patients were subject to coronary artery lesions, and the other 14 patients were classified as CALs- group. Patients with incomplete KD and other autoimmune diseases were beyond the scope of this study.

Treatment options involved the application of IVIG at a dose of 1 g/kg per day for two days and 30-50 mg/kg of oral aspirin per day from diagnosis establishment to defervescence. From each patient, one blood sample was obtained before treatment, and the other sample was collected after IVIG administration at which time the patient had not been febrile for at least 48 h. The healthy control samples were taken from children who came for healthy examination. None of these children had suffered any diseases in the previous month.

The Ethics Committee of The First Hospital of Jilin University approved the experimental protocol and it followed the guidelines of the Declaration of Helsinki. Written informed consents were obtained from all children’s parents. The recorded laboratory parameters from all individuals included: white blood cell counts, neutrophil counts, lymphocyte counts, serum C-reactive protein, erythrocyte sedimentation rate, and serum immunoglobulin (IgG, IgA, IgM).

### Flow cytometric analysis

In order to successful detection of cTfh cells, four milliliter fresh heparinized blood samples were collected separately from healthy controls and patients with KD both, before and after IVIG administration. Peripheral blood mononuclear cells (PBMCs) at 4 × 10^6^/ml were isolated from each individual at 800×g for 30 min at 25 °C by density-gradient centrifugation using Ficoll-Paque Plus (Amer- sham Biosciences, Little Chalfont, UK) and were stained with anti- CD3, anti-CD4, anti-CXCR5, anti-CD45RA, anti-CCR6, and anti-CXCR3 at room temperature for 30 min. BV510-conjugated anti-CD3, allophycocyanin (APC)-H7-conjugated anti-CD4, BB515-conjugated-anti-CXCR5, phycoerythrin (PE)-Cy5-conjugated anti-CD45RA, PE-Cy7-conjugated anti-CCR6, and APC-conjugated anti-CXCR3 were purchased from Becton Dickinson (San Jose, CA, USA). PBMCs were analyzed by multicolor flow cytometry (FACSAria II; BD Biosci-ences, Franklin Lakes, NJ, USA). Results were processed through FlowJo v10.0.7 software (Tree Star, Ashland, OR, USA).

### Measurement of cytokines levels

Levels of IFN-γ, IL-4 and IL-17A were measured by CBA Human Soluble Protein Master Buffer kit (BD Biosciences) using flow cytometer. We generated a standard curve for each set of reagents. The minimum and maximum detection limits for all six cytokines were 1.0 and 10,000 pg/ml, respectively. Quantification was performed using Cell- Quest Pro and CBA software (Becton Dickinson).

### Statistical analysis

The data are expressed as the median and range and analyzed with SPSS version 22.0 software. *P* < 0.05 is considered as statistically significant. Kruskal-Wallis test was used to analyze the difference among different phases of KD and healthy individuals as appropriate. Mann–Whitney test was applied to assess the difference between CALs+ and CALs- groups. The difference between before and after IVIG administration was analyzed using the Wilcoxon matched pairs test. The relationship between variables was evaluated using Spearman’s rank correlation test.

## Results

### Clinical parameters

The demographic and clinical characteristics of patients and controls are shown in Table 1. WBC, neutrophil counts, CRP and ESR were significantly higher in KD patients compared to controls. With the exception of CRP, that was significantly higher in CALs+ than in CALs-, WBC neutrophil counts and ESR had no significant difference between CALs+ and CALs-. Additionally, there were no significant differences in lymphocyte counts or serum immunoglobulin concentrations including IgG, IgA and IgM, neither between KD patients and controls or between CALs+ and CALs-.

**Table 1:** The demographic and clinical characteristics of the study participants

Parameters	Kawasaki disease (*n*=20)		Controls (*n*=15)
	CALs- (*n* = 14)	CALs+ (*n* = 6)	
Age (years)	3.2 (0.58–5)	2.8 (0.8–4.91)	2.95 (1.2–4.83)
Gender (Female/Male)	6/8	2/4	7/8
CRP (mg/L)	29.65 (3.14–151) ^#, *^	82.15 (34.4–186) ^#^	1.35 (0.75–3.23)
ESR (mm/h)	62.5 (11–110) ^#^	77.5 (30–130) ^#^	6 (3–13)
WBC (10^9^/L)	12.36 (6.99–31) ^#^	15.76 (5.89–23.3) ^#^	6.125 (4.78–9.5)
Neutrophils (10^9^/L)	9.135 (4–28.69) ^#^	11.5 (2.52–15.98) ^#^	2.42 (2.06–3.98)
Lymphocytes (10^9^/L)	2.765 (0.92–8.96)	3.825 (1.3–5.6)	3.038 (1.23–5.30)
IgG (g/L)	4.94 (1.82–14.1)	7.17 (6.15–9.08)	6.22 (2.08–9.37)
IgA (g/L)	0.575 (0.11–1.29)	1.01 (0.36–1.24)	1.08 (0.4–1.9)
IgM (g/L)	0.955 (0.29–1.96)	1.135 (0.37–1.42)	1.01 (0.62–1.9)

Data shown are median (range) or cases number. *CALs* coronary artery lesions, *CRP* C-reactive protein, *ESR* erythrocyte sedimentation rate, *Ig* immunoglobulin. *WBC* white blood cell counts. ^#^*P* < 0.05 vs. the Controls. ^*^P< 0.05 vs. CALs+ group

### Subsets of circulating Tfh cells and cytokine levels in different stages of KD

**Fig. 1:**
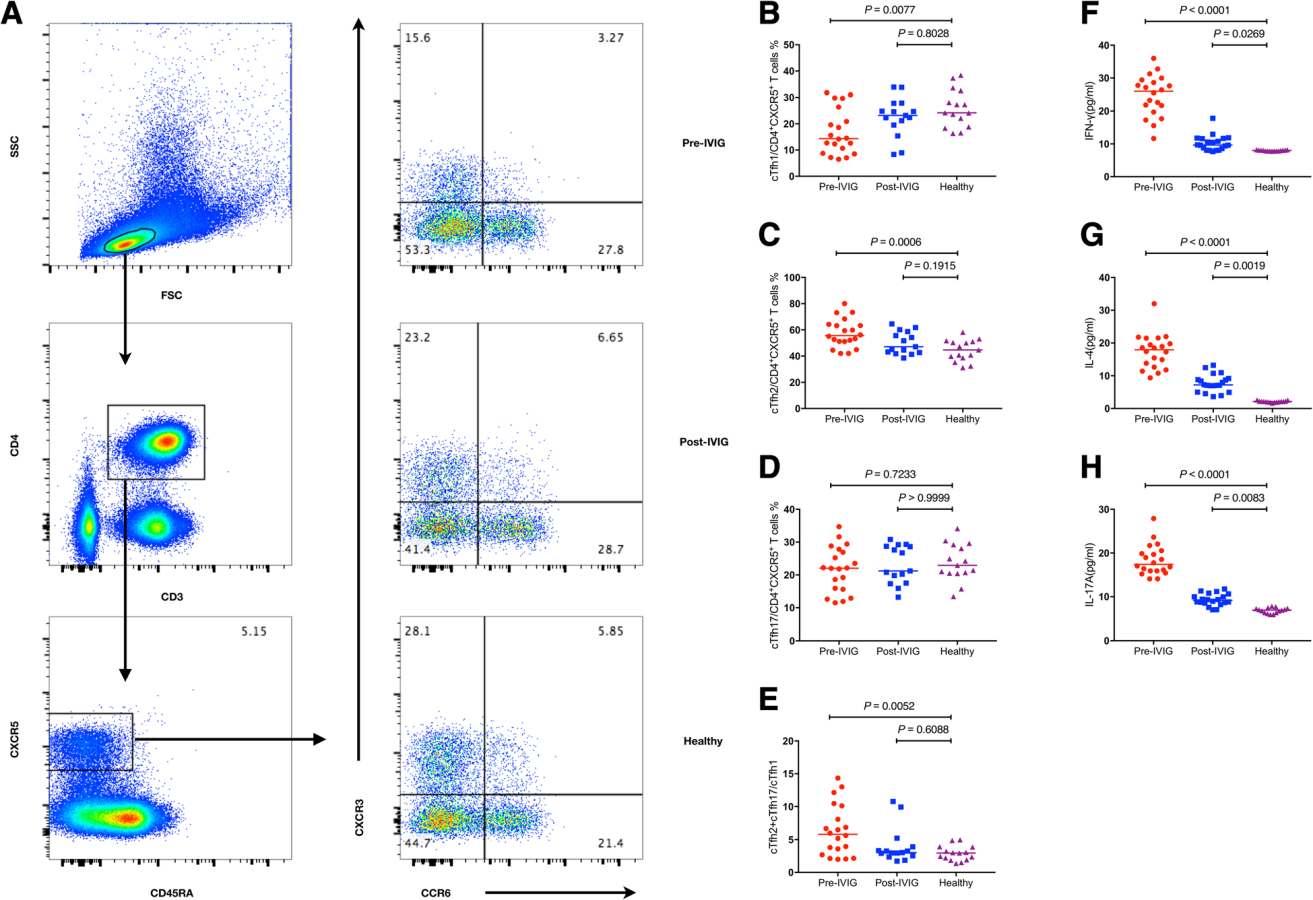
Flow cytometry analysis of the frequency of CD4+ T cells in KD patients. PBMCs from KD patients and control subjects were stained with fluorescent anti-CD3, anti-CD4, anti-CXCR5, anti-CD45RA-, anti-CXCR3 and anti-CCR6.The cells were gated initially on living lymphocytes, and then on CD3 + CD4+ T cells, and subsequently on CD45RA-CXCR5+ ^c^Tfh cells. The frequencies of CXCR3 + CCR6-, CXCR3-CCR6- and CXCR3-CCR6+ cTfh cell populations were analyzed by flow cytometry. **a** Flow cytometry analysis. **b**–**h** Quantitative analysis. Data shown are representative dot plots or are expressed as the percentage of cTfh cells of individual subjects. The horizontal lines represent the median values

To investigate the importance of cTfh-cell subsets in KD, PBMCs isolated from KD patients in different stages and HCs were immunostained for CD3, CD4, CXCR5, CD45RA, CD183 and CD196 and subsequently analyzed using flow cytometry. Upon the differential expression of CXCR3 and CCR6, three subsets were defined, CXCR3 + CCR6- Tfh (cTfh1) cells, CXCR3-CCR6- Tfh (cTfh2) cells and CXCR3-CCR6+ Tfh (cTfh17) cells, initially by gating on live lymphocytes, then on CD3 + CD4+ T cells and subsequently on CXCR5 + CD45RA- T cells (Fig. 1a). Before IVIG administration, percentage of cTfh1 cells was significantly lower compared to healthy subjects (*P* = 0.0077, Fig. 1b), whereas percentage of cTfh2 cells was significantly higher (*P* = 0.0006, Fig. 1c), and the variation of cTfh17 cells was not significant (*P* = 0.7233, Fig. 1d). As a result, the ratio of cTfh2 plus cTfh17 cells to cTfh1 cells significantly increased (*P* = 0.0052, Fig. 1e). Additionally, IFN-γ, IL-4 and IL-17A levels in KD patients were significantly higher compared to healthy controls (*P* < 0.0001, Fig. 1f; *P* < 0.0001, Fig. 1g; *P* < 0.0001, Fig. 1h). After IVIG administration, compared with healthy controls, there were no significant differences in the percentage of these three subsets. However, cytokine levels remained significantly higher compared to controls (*P* = 0.0269, Fig. 1f; *P* = 0.0019, Fig. 1g; *P* = 0.0083, Fig. 1h). Our data suggested that cTfh1 and cTfh2 cells, as well as these three cytokines, were involved in the pathogenesis of KD.

### The association among cTfh-cell subsets, cytokine levels and clinical parameters

**Fig. 2:**
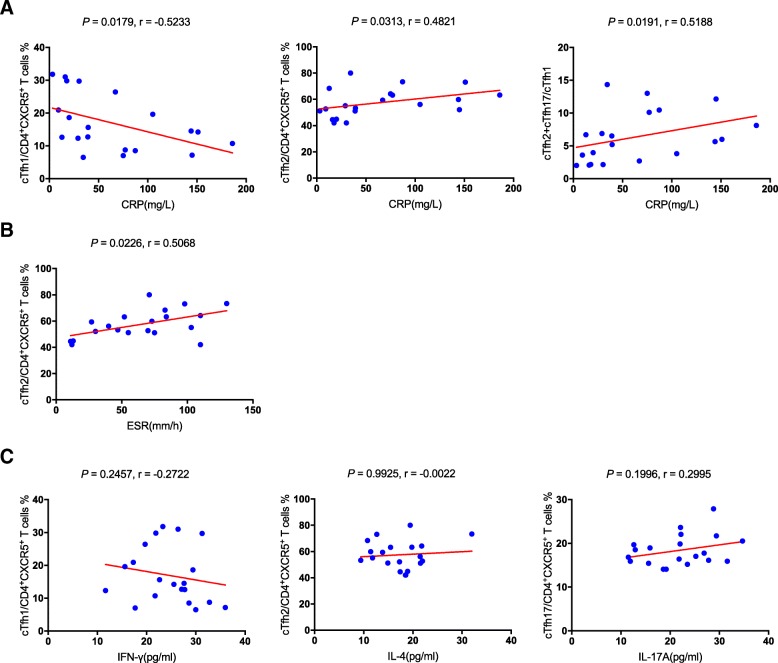
Correlation analysis. **a** The CRP values were negatively correlated with percentage of cTfh1 cells, and positively correlated with the percentage of cTfh2 cells and the ratio of cTfh2 plus cTfh17 cell to cTfh1 cells. **b** The ESR values were positively correlated with percentage of cTfh2 cells. **c** There were no correlations between cTfh1 cells and IFN-γ, cTfh2 cells and IL-4, or cTfh17 cells and IL-17A

To further addressed the role of cTfh cells in the pathogenesis of KD, we investigated the correlation among the distinct subsets of cTfh cells, clinical parameters such as CRP, ESR and serum immunoglobulin concentration, and cytokine levels including IFN-γ, IL-4 and IL-17A. The results (Fig. 2a) showed that percentage of cTfh1 cells was negatively correlated with the value of CRP (*P* = 0.0179, r = − 0.5233), whereas percentage of cTfh2 cells and the ratio were positively correlated with the value of CRP (*P* = 0.0313, r = 0.4821; *P* = 0.0191, *r* = 0.5188; respectively). Percentage of cTfh2 cells was also positively correlated with the value of ESR (*P* = 0.0226, r = 0.5068, Fig. 2b). Moreover, there was no correlation among cytokine levels and percentage of cTfh cells (Fig. 2c). None of other significant correlations was found, which suggested that decreased percentage of cTfh1 cells and increased percentage of cTfh2 cells corresponded to the high levels of CRP and ESR.

### Circulating Tfh-cell subsets and cytokine levels in different groups

**Fig. 3:**
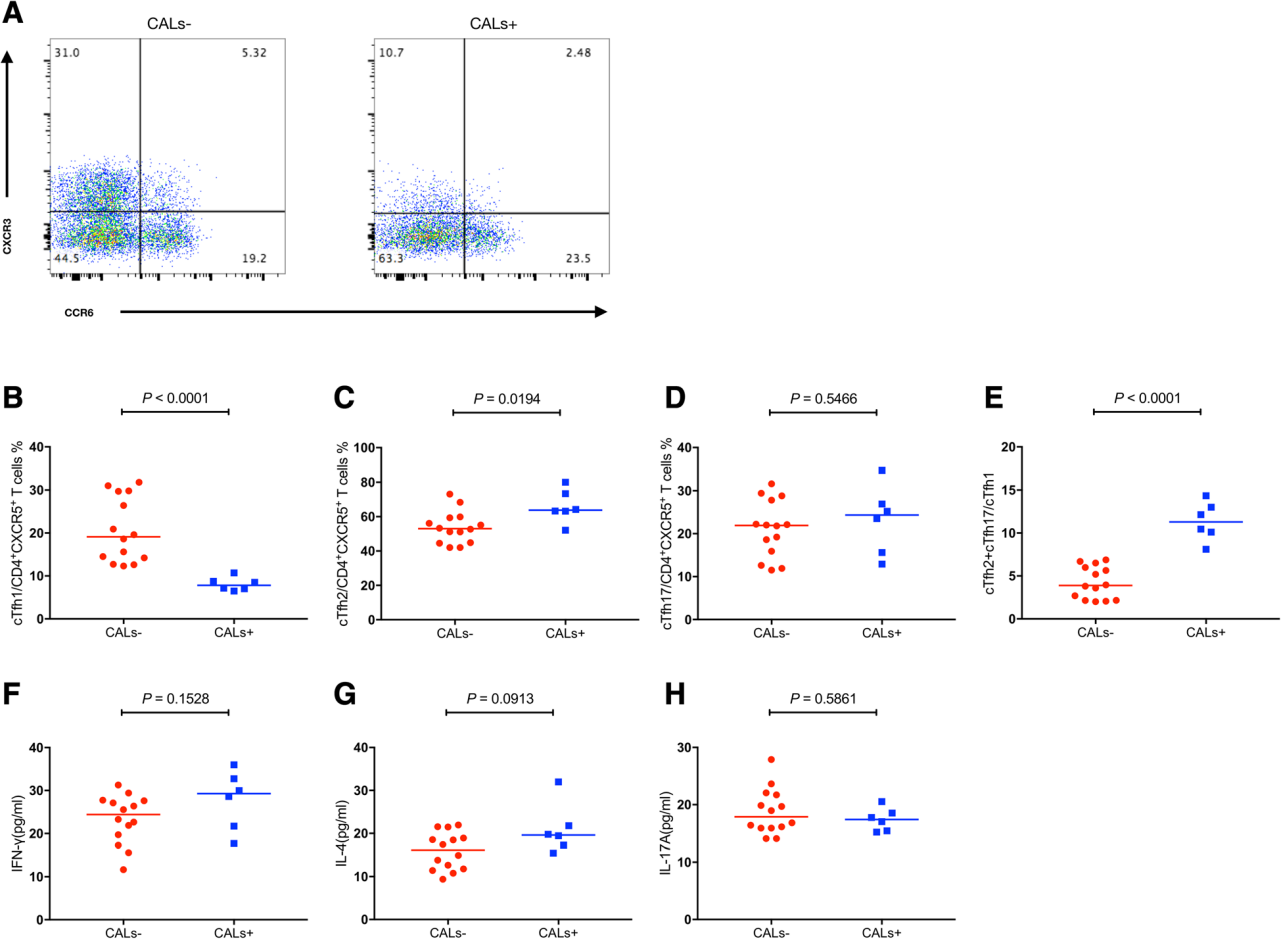
Flow cytometry analysis of the frequency of cTfh cells in KD patients with and without CALs. **a** Flow cytometry analysis. **b**–**h** Quantitative analysis. Data shown are representative dot plots or are expressed as the percentage of cTfh cells of individual subjects. The horizontal lines represent the median values. CALs, coronary artery lesions

Coronary artery lesions of KD lead to high mortality and disability rates. Consequently, understanding the immune-manifestation of CALs is beneficial for developing strategies for the management of KD patients. As shown in Fig. 3, the percentage of cTfh1 cells was significantly reduced in CALs+ group compared to CALs- group (*P* < 0.0001, Fig. 3b), whereas a significant elevated percentage of cTfh2 cells was observed in CAL+ group (*P* = 0.0194, Fig. 3c). Yet, there were no significant differences in the percentage of cTfh17 cells between two groups (*P* = 0.5466, Fig. 3d). With high expression of cTfh2 cells and low expression of cTfh1 cells, the ratio was significantly increased in CALs+ group (*P* < 0.0001, Fig. 3e). Differences in cytokine levels between two groups were not significant (Fig. 3f-h). Thus, much lower cTfh1 cells and higher cTfh2 cells might predict higher possibility of coronary artery lesions.

### Variation of cTfh-cell subsets and cytokines after treatment

**Fig. 4:**
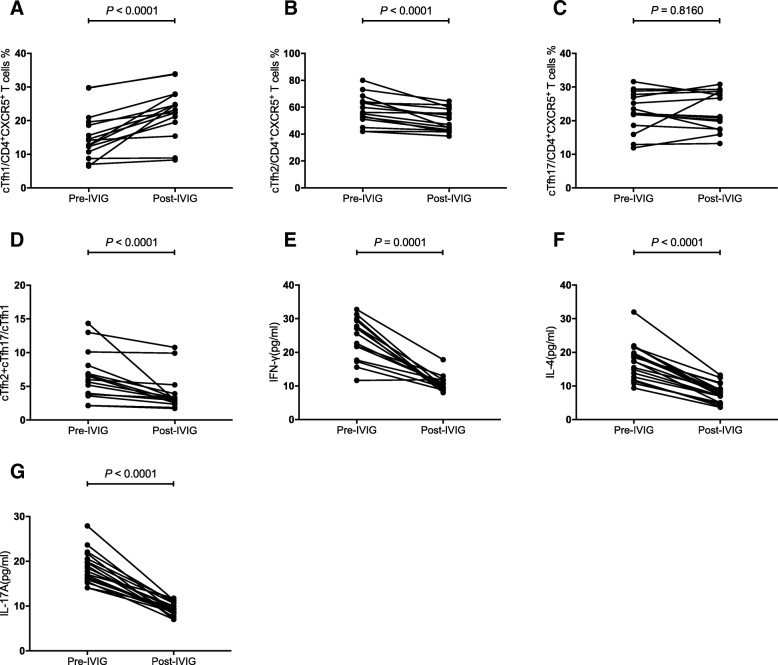
Variation in cTfh-cells subsets and levels of cytokines after IVIG administration. The frequency of the indicated cTfh cells and cytokine levels were compared between the pre and post IVIG administration

Next, we explored the percentages of distinct subsets of cTfh cells after IVIG treatment. Among these 20 KD patients, guardians of five patients, including two CALs+ and three CAL- patients, refused blood examination considering that clinical symptoms of their youngsters were improved. Regardless of the CALs+ or CALs- status, the patients showed a consistent variation trend. As exhibited in Fig. 4, percentage of cTfh1 cells significantly increased (*P* < 0.0001 Fig. 4a), whereas percentage of cTfh2 cells significantly decreased (*P* < 0.0001 Fig. 4b). Although the variation of percentage of cTfh17 cells was not significant (*P* = 0.8160, Fig. 4c), the ratio was significantly lower after IVIG application (*P* < 0.0001, Fig. 4d). Following clinical improvement, levels of cytokines were also downregulated (*P* = 0.0001, Fig. 4e; *P* < 0.0001, Fig. 4f; *P* < 0.0001, Fig. 4g). It can be concluded that the imbalance of cTfh-cell subsets with addition of high levels of IFN-γ, IL-4 and IL-17A contributed to the pathogenesis of KD patients, where following medical treatment, the aberrant immune state was restored.

## Discussion

CD4 + CXCR5+ T cells are well-known as a distinct subset of CD4+ T cells, which have the ability to induce B cell proliferation, to differentiate to plasma cells and to secret immunoglobulin. Tfh cells differentiate from naïve T cells via multi-stage and multi-factorial processes [20]. There is also another point of view according to which cTfh cells are derived from T helper cells because they are resemblant at transcriptional level [21]. Currently, the former conception is more convincible. Circulating Tfh cells are thought to be a memory compart of GC Tfh cells with the capacity of rapid and efficient secondary immune responses [22]. The contribution of cTfh cells in the pathogenesis of multiple diseases has been well-established. A study on the relation between cTfh cells and lupus has revealed an increased percentage of cTfh2 cells, associated with disease activity [23]. Another study has indicated that elevation of cTfh17 cells might participate in primary Sjögren Syndrome and that it may be used as biomarker for evaluating the activation of immune stage [24]. In our previous study, we have observed significantly increased cTfh2 and cTfh17 cells, which were related to the occurrence and development of IgA vasculitis [25]. Consequently, we concluded that cTfh cells have an essential role in autoimmunity. In the present study, percentage of cTfh1 cells significantly decreased before IVIG administration. Similarly, a previous research has shown reduced frequencies of IFN-γ-producing CD4+ and CD4- cells in KD [26]. On the contrary, the percentage of cTfh2 cells significantly increased in acute stage. In comparison with cTfh1 cells, cTfh2 cells display more efficient capacity in helping B cells and secreting IL-21 [12]. These data elucidate the increase of IL-21 in KD, which has been proved by Bae and colleagues [27]. It also can be speculated that there may be some changes in B cells. In fact, Yan Ding et al. found that both the absolute value and percentage of B cells in KD were significantly higher than those in healthy and febrile controls [28]. Besides, another study demonstrated that children with KD have a higher allergic susceptibility [29]. The elevated cTfh2 cells might contribute to this process due to their ability in secreting IL-4 and inducing IgE production. After applying IVIG, all these significant variations disappeared with clinical symptom improvement, which made us believe that this memory compartment was involved, even though the exact cause of KD remains unclear.

The over-represented cTfh2 cells and under-represented cTfh1 cells resulted in the imbalance of cTfh-cell subsets, which is thought to be related to disease activities such as SLEIDA score in SLE, PASI score in psoriasis and CDAI score in RA. However, the criterion for systemic assessment of KD activity remains to be established. CRP and ESR values are traditionally considered as the most commonly used indicators to evaluate inflammatory severity of KD. Through further investigation on the association between cTfh cells and CRP or ESR, we found that percentage of cTfh1 cells was negatively correlated with CRP, whereas percentage of cTfh2 cells was positively correlated with both CRP and ESR. Consequently, lower expression of cTfh1 cells and higher expression of cTfh2 cells corresponded to more sever inflammatory response. Interestingly, the existing research has demonstrated that patients with CRP levels higher than 30 mg/L and ESR higher than 40 mm/h are more likely to suffer coronary artery lesions [30]. Given that cTfh cells were correlated with CRP and/or ESR, which associated with CALs, we suspected that cTfh cells would be linked to CALs. So, next we compared percentages of distinct cTfh-cell subsets in CALs+ and CALs- groups and found much lower percentage of cTfh1 cells and much higher percentage of cTfh2 cells in CALs+ group. Therefore, cTfh1 and cTfh2 cells are essential in the development of CALs, and maybe potential indicators of CALs. An optional method is to formulate a range of cTfh1 and cTfh2 cells, which can predict the occurrence of CAL among KD patients, sequentially paying more attention to those individuals.

The role of cTfh1 and cTfh2 cells in KD may be related to their cytokines. CTfh1 cells have capacity to secret IFN-γ, which has been recognized to induce type I immunity through stimulating phagocytosis, the oxidative burst and production of proinflammatory cytokines [31]. Previous studies have shown that IFN-γ might contribute to activation of CD4 + CXCR3+ T cells and infiltration of these CXCR3+ T cells in the vessel wall [32]. Additionally, another experiment found that CD4 + CXCR3+ T cells infiltrated in the coronary arterial wall through autopsying dying patients during acute stage [33]. Thus, the percentage of cTfh1 cells in KD was significantly decreased, which might be explained with the expression of cTfh1 cells that is not a real down-regulation, but an increase in consumption. Based on these theories, reducing the level of IFN-γ and preventing these kinds of CD4 + CXCR3+ T cells from infiltrating the coronary arterial wall may be useful in the treatment of Kawasaki disease and prevention of CALs. By contrast, cTfh2 cells produce IL-4. IL-4 seems to have limited roles on the innate immune response. Nevertheless, IL-4 is characterized by inducing type-2 immunity, and is essential in mediating wound-repair, suppression of autoimmune disease and maintenance of tissue homeostasis [34]. Hence, it may be reasonable that the increase of cTfh2 cells and their correlation with CRP and ESR in KD are considered to be secondary to inflammatory to maintain the immune homeostasis. In summary, cTfh cells contribute to the development of KD not only by means of IL-21 secretion but also by producing other cytokines such as IFN-γ and IL-4, the levels of which were significantly higher in KD group. Significantly increased IFN-γ and IL-4 in acute stage of KD have also been proved by a Korean study, which has also indicated the importance of the two cytokines in KD [35]. However, neither our nor the Korean study found further elevated levels of IFN-γ or IL-4 in CALs+ group. We suspected that the formation of CALs, which is a complex process with too many unknown mechanisms that might include a decompensation signal pathway. Certainly, it is insufficient to explain the variation of cTfh cells only by these cytokines in KD patients with CALs. After all, the causes of CALs in KD are diversified, such as cell dysfunction, epigenetic hypomethylation and genetic polymorphisms [36, 37]. It is necessary to further investigate the relationship between cTfh cells and CALs+.

It was unexpected that both cTfh-cell subsets and the ratio were not correlated with the levels of serum immunoglobulin, which might be explained with the immature adaptive immune function in children. There are some limitations in this study that should be mentioned. Enlargement of the sample size, particularly of CAL+ group both before and after IVIG administration, is imperative. Moreover, IFN-gamma secreted by cTfh1 cells and IL-4 secreted by cTfh2 cells should be incorporated in the future studies.

## Conclusions

In conclusion, to the best of our knowledge, this is the first study that described these three subsets and their imbalance in KD patients. Among these subsets, cTfh1 and cTfh2 cells might have a key role in the development of KD as well as in progression of CALs. It might be beneficial to keep the balance of cTfh-cells subsets for preventing CALs.

## Abbreviations

CALs: Coronary artery lesions
cTfh cells: Circulating T follicular helper cells
GC: Germinal center
IVIG: Intravenous immunoglobulin
KD: Kawasaki disease
PBMC: Peripheral blood mononuclear cells
